# Supplementary figures and images for: CD73 Promotes Tumor Progression in Patients with Esophageal Squamous Cell Carcinoma
Source: Cancers (Basel). 2021 Aug 9;13(16):3982. doi: 10.3390/cancers13163982 (PMC8393769; doi:10.3390/cancers13163982)

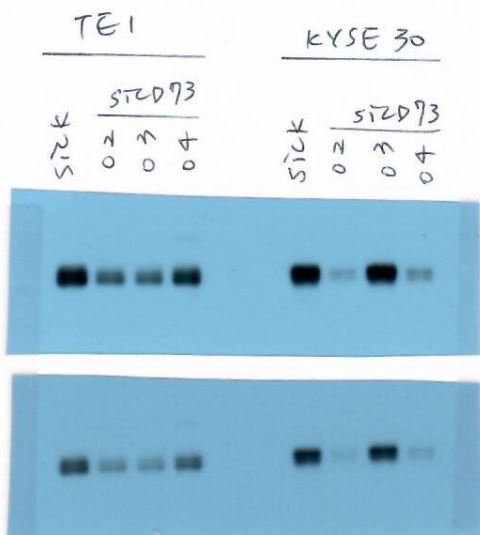

CD73.

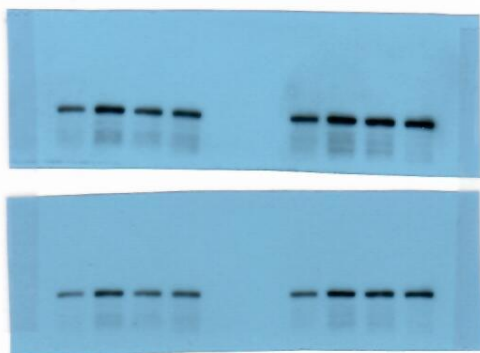

E-cadherin

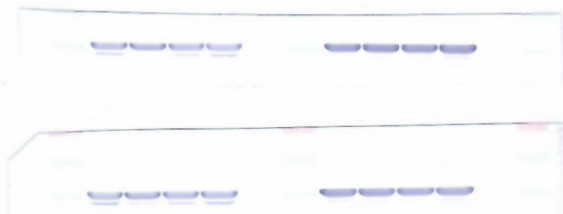

Actin

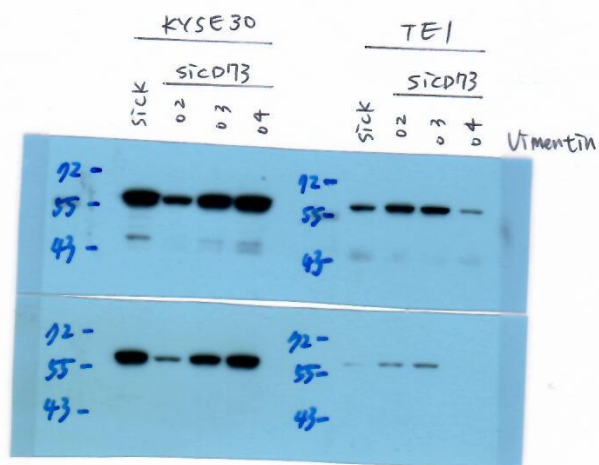

Vimentin

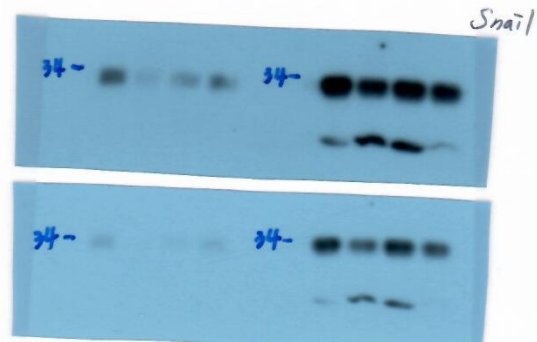

Snail

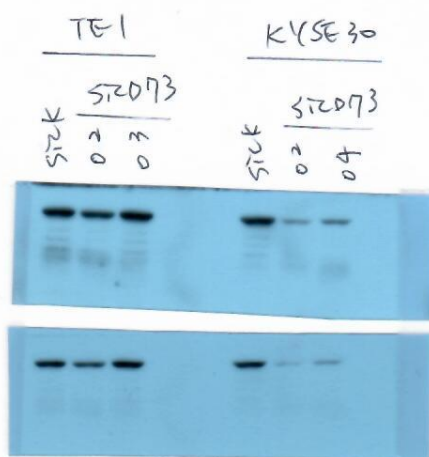

Vimentin

Supplement: Supplementary file 1 [file cancers-13-03982-s001.zip › cancers-1309529-supplementary.pdf]
